# A case report of an expensive yet necessary thoracentesis: Expanding the boundaries of endoscopic ultrasound transbronchial needle aspiration

**DOI:** 10.1097/MD.0000000000017555

**Published:** 2019-10-11

**Authors:** Steven Cocciardi, Amit Borah, Rocco Terrigno, Wissam Abouzgheib, Ziad Boujaoude

**Affiliations:** Division of Pulmonary and Critical Care Medicine, Cooper Medical School of Rowan University, Cooper University Hospital, Camden, NJ.

**Keywords:** EBUS, pleural effusion, thoracentesis

## Abstract

**Rationale:**

Endobronchial ultrasound has revolutionized the field of bronchoscopy and has become one of the most important tools for the diagnosis of intrathoracic lymphadenopathy and para-bronchial structures. The reach of this technique has not been limited to these structures and pleural lesions have been at times accessible. To our knowledge, pleural fluid collections have not been accessed with endobronchial ultrasound (EBUS).

**Patient concerns:**

52-year-old women with dyspnea, fever and a new loculated pleural effusion that was suspected to be the source of the fever but was not accessible through traditional thoracentesis.

**Diagnosis:**

Malignant pleural effusion.

**Interventions:**

Sampling and drainage of the loculated pleural fluid collection using EBUS scope introduced via the esophagus.

**Outcomes:**

Infection excluded. Resolution of fever and improved dyspnea after drainage of effusion.

**Lessons:**

The convex curvilinear ultrasound bronchoscope allows unprecedented access to thoracic structures. The reach is not limited to mediastinal lymph nodes and parenchymal masses adjacent to the airways, and pleural space and pleural fluid are at times accessible, particularly when one considers the esophageal approach.

## 1 Introduction

Since its introduction in 2004, endobronchial ultrasound-transbronchial needle aspiration (EBUS-TBNA) has become one of the most important tools available to chest physicians for the diagnosis of intrathoracic lymphadenopathy and para-bronchial structures.^[1]^ The reach of this technique is not limited to these structures, and cases of pleural lesion biopsies and transvascular biopsies have been reported^[2,3,4]^ To our knowledge, pleural fluid collections has not been accessed with EBUS. We present a case of a loculated fluid collection that was suspected to cause fever and dyspnea and was not amenable to traditional thoracentesis, but was successfully sampled and drained using the EBUS scope via the esophagus.

## 2 Case presentation

**Figure 1:**
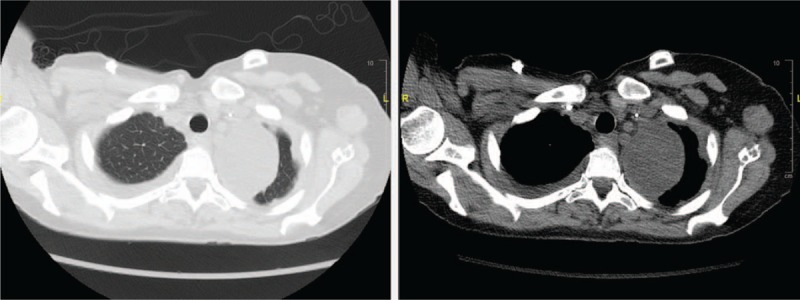
Left apical and medial pleural fluid collection.

**Figure 2:**
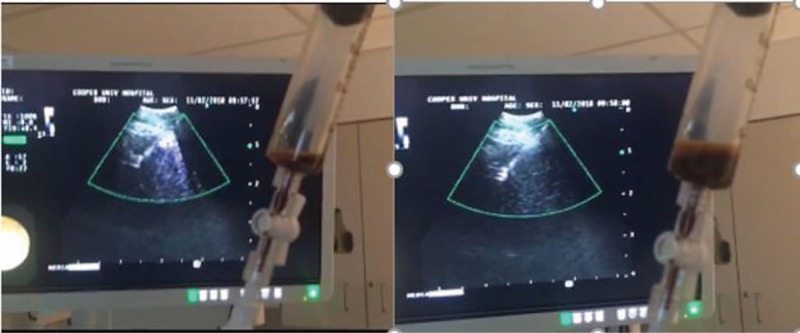
Transesophageal needle aspiration with EBUS scope. EBUS = endobronchial ultrasound.

A 52-year-old woman with a history of stage 4 breast cancer presented to the hospital for fever of 1-week duration and worsening shortness of breath. She had known pleural involvement of her cancer and underwent tunneled pleural catheter placement 8 months prior due to recurrent malignant pleural effusion. She achieved spontaneous pleurodesis and the catheter was eventually removed without significant residual fluid collection seen on imaging. Upon our exam, the prior tunneled catheter site was nonerythematous and clearly not infected. The chest x-ray; however, showed a new left apical well-rounded opacity. Subsequent chest computed tomography scan showed a new 6.5 cm well-circumscribed left apical lesion of homogeneous density consistent with fluid as opposed to a mass (Fig. 1). The patient was started on broad-spectrum antibiotics but continued to have fevers. Due to persistent fevers without a clear source despite extensive work-up, our team pursued sampling and drainage of this collection. Given the apical and medial location with the proximity to the esophagus, we used the EBUS bronchoscope via an esophageal approach to access the fluid collection (Fig. 2). One hundred fifty cc of dark brown fluid was drained successfully, and the patient tolerated the procedure well. The patient's fever resolved the following day, her dyspnea improved, and she was discharged home and completed a 14-day course of antibiotics. Fluid analysis was consistent with an exudate with slightly decreased glucose. No organisms grew from the pleural fluid, possibly due to several days of antibiotics before the procedure. Final fluid cytology was positive for malignancy consistent with known patient malignancy.

## 3 Discussion

The convex curvilinear ultrasound bronchoscope (EBUS scope) has revolutionized the field of bronchoscopy and has become one of the most important tools available to chest physicians for the diagnosis of intrathoracic lymphadenopathy, para-bronchial structures and the staging of lung cancer.^[1]^ It is recommended as a first step sampling technique by the American College of Chest Physicians 2013 evidence-based practice guidelines.^[5]^ The reach of the EBUS scope has even extended beyond these structures and cases of pleural lesion biopsies and transvascular biopsies have been described.^[2,3,4]^ The applications of this scope are not; however, limited to a bronchial approach, and the EBUS scope has been used via the esophagus for the diagnosis of nodal and non-nodal thoracic abnormalities. It is particularly effective for left para-aortic, para-esophageal, and sometimes aortopulmonary window lymph node stations. It is also effective for some left upper lobe parenchymal masses and sometimes for sampling of the left adrenal gland. The use of the esophagus is often a better option for some patients with limited respiratory reserve and sometimes the only available option, such as the case of a patient intubated with a small endotracheal tube.^[6,7]^ To our knowledge, this is the first description of pleural fluid aspiration using an EBUS scope via the esophagus. It did allow providing a diagnosis and a treatment that was not otherwise possible. Certainly, the location of this patient's pleural collection was favorable for an esophageal approach. While this may not be the case in many pleural collections, this approach should be considered when the circumstances are favorable.

A legitimate concern is the risk of introducing an infection in the pleural space. EBUS-TBNA has been shown to be a safe procedure with very low infectious complications rate. Most infectious complications were reported when the puncture site is necrotic or cystic, where the blood flow is slight, resistance is decreased, and bacterial attachment clearance is decreased.^[8,9]^ This is of real concern when puncturing a pleural fluid collection. The postulated mechanism for pleural inoculation is that as the bronchoscope traverses the naso-oropharyngeal region, the working channel becomes contaminated. When the transbronchial needle passes through the working channel, the needle becomes contaminated and could potentially inoculate sampled tissue. Although the needle designed for this scope has an outer sheath to minimize sample contamination, this sheath still passes through the working channel. When the needle is deployed at the site of interest, it passes through the distal end of this sheath and can become contaminated. Another possibility is contamination of the stylet when multiple punctures are applied. To prevent these complications, universal guidelines should be followed including disinfection of devices according to the guidelines and careful observation of the post-procedure course for signs of infection are important.^[10]^ Particular attention should be given to avoid stylet contamination. Prophylactic antibiotic administration, although not recommended in routine diagnostic bronchoscopy, could be considered when puncturing fluid collections and cystic lesions. In our case, in addition to our patient being on antibiotics, attention was paid to stylet manipulation and the number of punctures was limited.

In conclusion, the convex curvilinear ultrasound bronchoscope allows unprecedented access to thoracic structures. These structures are not limited to mediastinal lymph nodes and parenchymal masses adjacent to airways, and pleural space and pleural fluid are at times accessible. Additionally, an esophageal approach to making the diagnosis sometimes offers opportunities not permitted via the airways, either due to anatomy or greater patient risk. We hope that this case provides an insight into the utility of this tool in the hands of the interventional pulmonologist.

## Author contributions

**Conceptualization:** Steven Cocciardi, Ziad Boujaoude.

**Data curation:** Steven Cocciardi, Ziad Boujaoude.

**Formal analysis:** Ziad Boujaoude.

**Writing – original draft:** Steven Cocciardi, Ziad Boujaoude.

**Writing – review and editing:** Amit Borah, Rocco Terrigno, Wissam Abouzgheib, Ziad Boujaoude.

## References

[1] Hwangbo B Lee GK Lee HS Transbronchial and transesophageal fine-needle aspiration using an ultrasound bronchoscope in mediastinal staging of potentially operable lung cancer. Chest 2010;138:795–802. 20348194 10.1378/chest.09-2100

[2] Boujaoude Z Pratter M Abouzgheib W Transpulmonary artery needle aspiration of hilar masses with endobronchial ultrasound: a necessary evil. J Bronchol Intervent Pulmonol 2013;20:349–51. 10.1097/LBR.0000000000000011 24162122

[3] Gaspard D Raja H Arya R A case report on the diagnosis of a rare pleural tumor with endobronchial ultrasound: breaking new boundaries. Medicine 2015;94:e561. 25761175 10.1097/MD.0000000000000561 PMC4602464

[4] Panchabhai TS Machuzak MS Sethi S Endobronchial ultrasound-guided transvascular needle aspiration: a single-center experience. J Bronchology Interv Pulmonol 2015;22:306–11. 26492603 10.1097/LBR.0000000000000227

[5] Silvestri GA Gonzalez AV Jantz MA Methods for staging non-small cell lung cancer: diagnosis and management of lung cancer, 3rd ed: American College of Chest Physicians evidence based clinical practice guidelines. Chest 2013;143 Suppl 5:e211S–50S. 23649440 10.1378/chest.12-2355

[6] Abouzgheib W Shweihat Y Bartter T Oesophageal applications of the convex curvilinear ultrasound bronchoscope; an illustrative case series. Respirology 2011;16:965–8. 21564402 10.1111/j.1440-1843.2011.01991.x

[7] Meena N Hulett C Jeffus S Left adrenal biopsy using the convex curvilinear ultrasound scope. Respiration 2015;89:57–61. 25502409 10.1159/000368370

[8] Eapen GA Shah AM Lei X American College of Chest Physicians Quality Improvement Registry, Education, and Evaluation (AQuIRE) Participants. Complications, consequences, and practice patterns of endobronchial ultrasound-guided transbronchial needle aspiration: Results of the AQuIRE registry. Chest 2013;143:1044–53. 23117878 10.1378/chest.12-0350 PMC3616680

[9] Parker KL Bizekis CS Zervos MD Severe mediastinal infection with abscess formation after endobronchial ultrasound-guided transbrochial needle aspiration. Ann Thorac Surg 2010;89:1271–2. 20338352 10.1016/j.athoracsur.2009.09.002

[10] Mehta AC Prakash UB Garland R American College of Chest Physicians and American Association for Bronchology consensus statement: prevention of flexible bronchoscopy-associated infection. Chest 2005;128:1742–55. 16162783 10.1378/chest.128.3.1742 PMC7094662

